# Thermal Image Processing for Respiratory Estimation from Cubical Data with Expandable Depth

**DOI:** 10.3390/jimaging9090184

**Published:** 2023-09-13

**Authors:** Maciej Szankin, Alicja Kwasniewska, Jacek Ruminski

**Affiliations:** 1 Intel Corporation, 16409 W Bernardo Dr Suite 100, San Diego, CA 92127, USA; 2 SiMa Technologies, 226 Airport Pkwy Ste 550, San Jose, CA 95110, USA; alicja@sima.ai; 3 Department of Biomedical Engineering, Gdansk University of Technology, Gabriela Narutowicza 11/12, 80233 Gdansk, Poland; jacek.ruminski@pg.edu.pl

**Keywords:** convolutional models, increased receptive field, neural networks, regression, thermal imagery

## Abstract

As healthcare costs continue to rise, finding affordable and non-invasive ways to monitor vital signs is increasingly important. One of the key metrics for assessing overall health and identifying potential issues early on is respiratory rate (RR). Most of the existing methods require multiple steps that consist of image and signal processing. This might be difficult to deploy on edge devices that often do not have specialized digital signal processors (DSP). Therefore, the goal of this study is to develop a single neural network realizing the entire process of RR estimation in a single forward pass. The proposed solution builds on recent advances in video recognition, capturing both spatial and temporal information in a multi-path network. Both paths process the data at different sampling rates to capture rapid and slow changes that are associated with differences in the temperature of the nostril area during the breathing episodes. The preliminary results show that the introduced end-to-end solution achieves better performance compared to state-of-the-art methods, without requiring additional pre/post-processing steps and signal-processing techniques. In addition, the presented results demonstrate its robustness on low-resolution thermal video sequences that are often used at the embedded edge due to the size and power constraints of such systems. Taking that into account, the proposed approach has the potential for efficient and convenient respiratory rate estimation across various markets in solutions deployed locally, close to end users.

## 1. Introduction

In recent years, there has been an unprecedented surge in demand for remote healthcare services. Apart from the most evident cause of this change, i.e., the pandemic [1], this tendency can also be attributed to multiple health-related trends observed in modern society. These trends include but are not limited to aging societies [2] followed by more effort being put into preventing ageism and digital exclusion [3], along with advances in wearable devices promoting more healthy lifestyles [4]. At the same time, advances in artificial intelligence made vision systems more robust and feasible than ever [5].

Given all of the above, it is of the utmost importance to develop a solution that would enable remote medical diagnosis that is both precise and convenient. The latter is an important aspect, as a convenient solution is used more frequently and thus can provide necessary feedback to help shape a healthier lifestyle and identify anomalies faster. Unarguably, one of the core metrics of vital signs estimation is respiratory rate (RR) [6]. However, most currently employed solutions require a professional to set up the equipment and/or perform measurements while maintaining physical contact with a patient [7], which can be disruptive and discourage from being used more often. Additionally, all solutions that require skin contact are heavily regulated, leading to an extended time-to-market due to a required certification and, thus, also an increased development cost. This reasoning highlights the importance of developing contactless solutions that would allow for providing cost-efficient systems that could be quickly deployed at home or public spaces, such as entries to buildings or border control stations.

The issue of vital sign estimation has recently become an area of increased interest from researchers. While proposed solutions are often contactless, they usually rely on the visible light spectrum (380 to 700 nm electromagnetic radiation wavelength) [8]. The visible light spectrum can be captured with commodity hardware, such as an off-the-shelf USB web camera or a small-form-factor MIPI camera module that can be added to a compute board. While these sensors are relatively inexpensive, they may suffer from variable environmental conditions, such as sensitivity to lightning or climatic changes (rain, fog). Systems based on visible light sensors also have poor adaptability to variations in skin tone and other facial features, rendering them more cumbersome to use across the entire population. As was reported in [9], test subjects belonging to groups IV–VI on the Fitzpatrick scale have an estimated 25% higher error rate than the other groups, as tested with the task of face detection on the CCD [10] dataset. Furthermore, visible light sensors also do not protect patients’ privacy, as they capture high-fidelity features making it easy to identify the person, and the action being performed along with the background information. The use of thermal cameras has been researched to address the shortcomings of solutions based on the visible light spectrum. Popular thermal cameras operate in long-wave infrared (LWIR) range, sometimes called “far-infrared”, typically between 8 to 12 µm. However, this was only done in a limited scope due to the high cost and relatively poor quality/resolution of images. To solve the latter, some researchers have been evaluating the influence of the Super Resolution algorithms on the accuracy of the neural-network-based predictions using thermal video sequences with the quality enhanced during the pre-processing step [11].

In view of the foregoing, this study focuses on the feasibility of an end-to-end convolutional model for an RR estimation task without the need for additional calibration, signal extraction, or data conversion to the frequency domain that often requires higher numerical precision. In addition, the deep learning model used in the study operates directly on low-resolution thermal images, which usually have lower resolutions than their visible light counterparts and it is not uncommon to see resolutions of 160 × 120 or even 80 × 60, without the need for applying additional image enhancement techniques. Therefore, the proposed method has a potential for deployment in practical systems, because such low-resolution sensors are smaller and much more affordable, making them suitable for embedded edge processing in home monitoring, epidemiological diagnostics, and other contactless health state estimation systems, e.g., at airports. Specifically, this study builds on top of the SlowFast model [12] used for an action recognition task in video streams to perform the RR task from low-resolution thermal images. The main idea behind the SlowFast model is to combine two streams of information with different temporal resolutions. Each stream is processed by a separate branch within the model, which allows for an accurate representation of both spatial and temporal features in videos. Building upon this methodology, a similar approach might be suitable for the contactless estimation of vital signs with thermal images. Similarly to the action recognition task, a unique sampling frequency is required for both feature detection and regression. Thus, this work proposes a fully convolutional model for Respiratory Estimation from Cubical Data with Expandable Depth (RESCUED), where the Expandable Depth corresponds to different window sizes used for capturing signals and varying sampling frequency for two paths of the model. In particular, our contributions are three-fold:

1. Slow and Fast branches are adapted to capture breathing signals by changing the sampling frequencies. Specific rates are selected based on the extensive grid search of the network parameters (Section 3.1).
2. Convolutional filters used for feature extraction and for capturing temporal dependencies between signal frames are modified to increase the receptive field and capture more distant dependencies between features (Section 3.2).
3. Finally, the classification head is replaced by a regression layer to output RR values instead of the recognized action category (Section 3.3).

The rest of the work is structured as follows: Section 2 describes the related work in contactless vital sign estimation techniques, with a specific focus on thermal data processing. Section 3 provides details of the proposed convolutional-based network for RR estimation. Experimental analysis is discussed in Section 4, along with the preliminary results, further summarized in Section 5. Finally, Section 6 concludes the study and provides directions for further enhancements.

## 2. Related Work

Thermal imaging has already been successfully used to evaluate human RR from a sequence of facial images; however, most of the methods are still multi-step procedures, requiring tuning of each phase separately, which is time-consuming and may affect final performance both in terms of accuracy and computational overhead [13]. Such systems are usually based on three data processing phases: (1) manual or automatic identification of an ROI and extraction of the respiratory signal using a chosen aggregation operator for each ROI datum (e.g., mean [14], skewness [15]), (2) signal pre- or post-processing to improve its quality (e.g., Hampel and bandpass filters [16], moving-average filtration [14], etc.), (3) identification of respiratory signal parameters such as RR (e.g., peak detection [17], dominating peak in the frequency domain [18], wavelet analysis [19], auto-correlation techniques [20]), or other respiration-related properties (e.g., respiratory patterns [18]). Several studies (e.g., [17,21,22]) address the practical aspects of RR estimation using cost-effective but low-resolution thermal cameras that were introduced to the market, such as Tamarisk, FLIR Lepton, or similar camera modules. Some methods are proposed to introduce the additional pre-processing step for improving the quality of input sequences, thus enhancing the dynamics of the pixel color changes used for vital sign extraction. These methods are based on color magnification [23], super-resolution—including Convolutional Neural Network (CNN)-based DRESNet [11] and Transformer-based TTSR [5], and denoising [24] with computer vision and deep neural network approaches. Studies [25,26] show examples of such a multi-step procedure. The camera observed the side view of the subject, and the recorded frames were used to visualize the breathing dynamics. Then, the signal processing algorithm was applied, using a narrow band-pass filter in the CO${}_{2}$ absorption band (4.3 µm) to obtain the CO${}_{2}$ content of exhaled air during breathing activity. Ground-truth measurements were collected using an abdominal transducer for nine subjects. The results showed a high correlation between the thermal-based and ground-truth breath rates.

Most of the later works used a thermal camera positioned collinearly to the subject’s face. The surface temperature changes are observed near the nostrils or/and the mouth during inhalation and exhalation of air. Typically, the respiratory signal is extracted using aggregation of the useful data from each frame into a sequence of values that form an estimated respiratory signal. In [27], a multi-step process consisting of signal averaging, decomposition, and filtering was applied before analysis of the dominant peak in the frequency spectrum corresponding to the RR, comparing the results with the ground truth data obtained for infants using an electrocardiogram. A similar approach was used in [28]. The signal was constructed using the mean of pixel values in the nostril area and filtered using the band-pass filter. Next, similarly, to other studies, the Fourier transform was used to calculate the power density spectrum of the filtered respiration signal, and the dominant peak was used for RR estimation. Innovation of the proposed method lies in 3D face alignment for accurate nostril tracking and thus improved RR estimation accuracy. However, that step requires additional processing and makes the method more complex.

In fact, most methods vary in the selection and tracking of the Region of Interest (ROI), as the rest of the algorithm, the RR estimation, is most commonly solved using similar algorithms based on analysis of the frequency spectrum. The proper identification of the ROI as a source of the thermal-based respiratory signal is key in such solutions. The expert-based, manual method can be used for a few cases [29], but cannot be used for real-time processing. A semi-automatic facial mark system integrating human knowledge within the multi-scale facial marks was proposed in [30], proving that geometric distributions of facial features can be used for face analysis. A similar concept was also utilized in [31], where authors took advantage of human anatomy and face geometry for remote monitoring of breathing dynamics using infrared thermography. Recently, even more automation was introduced to the ROI selection process, e.g., methods based on the detection of minimum and maximum color intensities followed by conventional image segmentation [32].

In addition to ROI detection, it is also crucial to track the nostril area in case of head movements. Various tracking methods have been already proposed in the literature, also to extract an optimized respiratory-related signal (e.g., [28,33,34]). Due to the recent advances in deep learning, such methods have been also already proposed to detect the face, nose, or mouth in thermal images as potential ROIs for extraction of respiratory signals [17,35,36]. Although many techniques have already been proposed for contactless vital signs estimation [8], the majority of them still use a combination of various algorithms for RoI detection, signal extraction, and respiratory estimation, e.g., a convolutional model followed by clustering [37]. To the best of our knowledge, the task of RR estimation using thermal sequences has not been solved yet using a single-shot approach, where the RR value would be directly predicted with a feed-forward deep neural network given a sequence of thermal recordings. This work aims to address this gap with a 3D convolutional model. Such an approach is unique compared to previous methods, which require multiple steps combining neural networks with multiple image and signal processing algorithms.

## 3. Methodology

**Figure 1:**
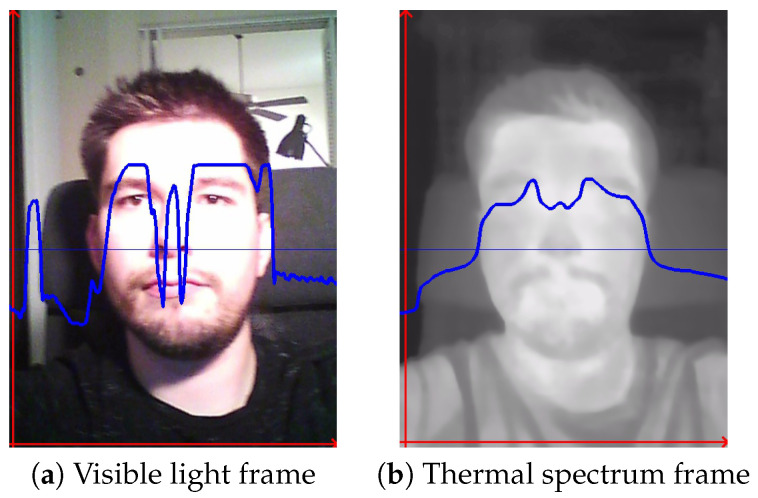
Visualization of pixel value dynamics across two domains—visible (**a**) and thermal (**b**) spectrum. To keep this comparison comprehensive, only neighboring pixels from a single dimension were used, as indicated by the thin horizontal blue line. The thick blue line represents a scale of differences between neighboring pixels at the same point on the horizontal line.

**Figure 2:**
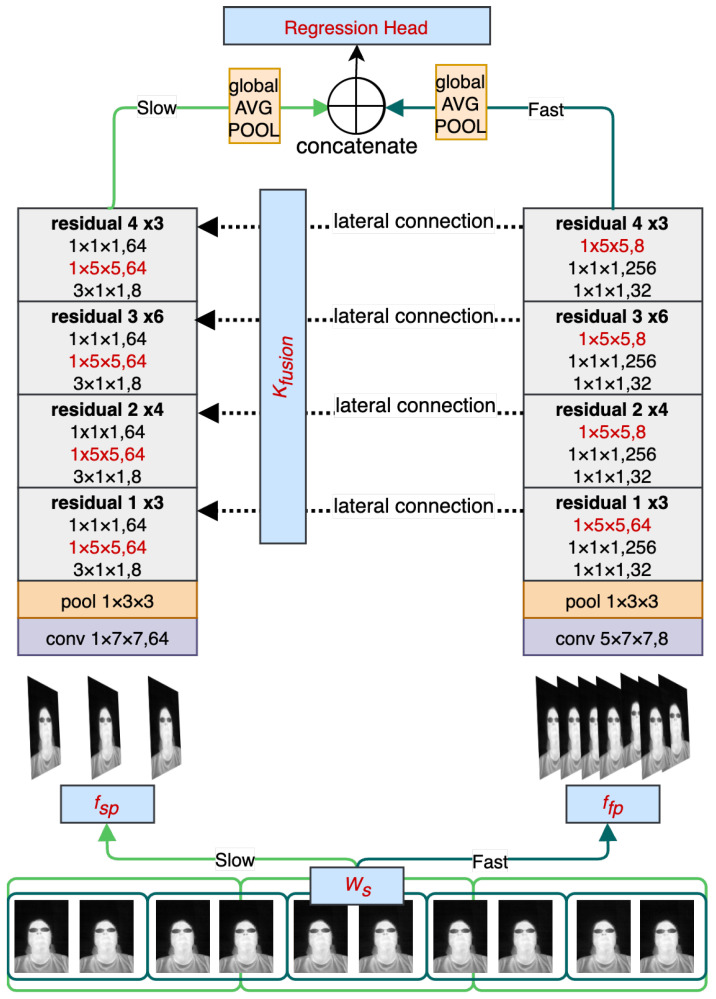
High-level overview of the proposed RESCUED model. Blocks marked with red font are the custom blocks introduced in the proposed model: $W_{s}$—Sliding window size; $f_{SP}$/$f_{FP}$—sampling frequency for Slow and Fast Paths; $r_{F}$—receptive field; $K_{Fusion}$—kernel size of the convolution used to fuse from Fast pathway to Slow pathway.

The main objective of RESCUED is to verify if the convolutional model originally designed for action recognition from visible light video sequences could be repurposed for the task of RR estimation from low-resolution thermal sequences. It is important to note that the characteristics of data from both domains significantly differ, and thus a direct application of such a network might be challenging and require topology modification. Visible light data contain high-frequency components, such as sharp edges and higher contrast changes in adjacent regions. On the other hand, thermal data are characterized by blurriness, and changes are less dynamic in values of the spatial representation due to heat flow in objects, as can be seen in Figure 1. Following findings previously discussed in the Super Resolution models targeting thermal data [35], this work introduces three main new features to the established state-of-the-art action recognition SlowFast model [12] to obtain RR values using the one-shot approach. The high-level view of the model is shown in Figure 2. It consists of two branches with varying sampling frequencies that can cover faster changes corresponding to facial expressions and face movements (Fast path) and slower changes corresponding to changes in the temperature of inhaled and exhaled air during breathing episodes. Receptive fields in both branches have been modified to address more distant relations between adjacent regions in thermal images caused by the heat flow in objects. In addition, the head of the model has also been modified by removing the fully connected layer and replacing it with the regression output corresponding to estimated RR values. Each of the contributions is described in more detail in the following subsections.

### 3.1. Sampling Rate and Fusion Kernel

The motivation for sampling rate modification is explained by introducing the details of SlowFast architecture first. The SlowFast model was designed for action recognition tasks, where the Slow branch is responsible for detecting objects involved in the action, given the fact that the type of objects is usually persistent across visible changes, and the Fast branch is used for action recognition with a relatively high sampling rate to be able to capture fast and sudden movements. Respiratory events have different characteristics. First, the respiratory signal is periodic, meaning that the dynamics of respiration are consistent across the window used to capture the signal. Secondly, the respiratory signal’s period is longer than the length of the activity stored in the dataset for the action recognition models [38].

**Figure 3:**
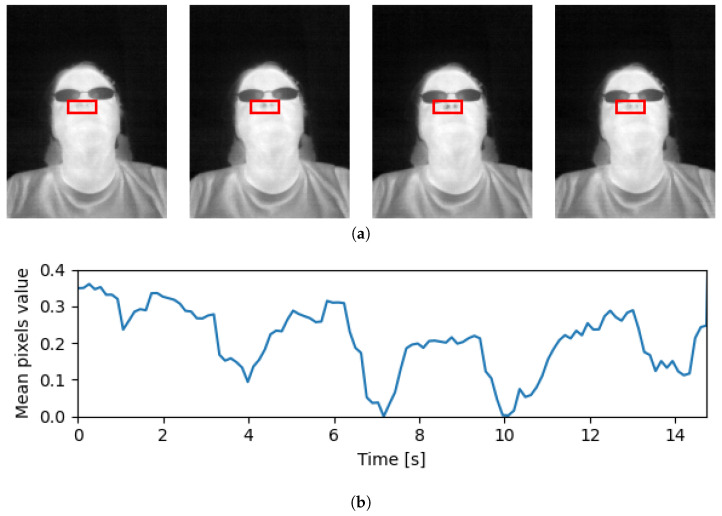
An example of a subset of frames extracted from a thermal video sequence with a visible change in temperature for the nasal region caused by inhalation and exhalation along with a plot of mean color change for this region over time. (**a**) Sequence of frames captured in thermal spectrum showing a full breathing episode. The red rectangle highlights the nasal region for which the signal was extracted. (**b**) Changes in average pixel values for the tracked nasal region shown in (**a**). This particular sequence (**b**) has 6 inhales and 5 exhales, as indicated by peak and valley points on the plot, respectively.

A breathing episode is a term used to refer to a complete cycle of inhalation and exhalation that occurs during the process of breathing. The duration of a breathing episode may vary depending on a number of factors, including the individual’s RR, lung capacity, and physical activity level. Thermal vision sensors allow for capturing of breathing episodes by analysis of temporal changes in pixel values corresponding to differences in temperatures inhaled and exhaled air, as presented in Figure 3. Such changes can be captured using the three-dimensional convolutional filters, where the depth of the filter corresponds to the temporal temperature change.

Therefore, the original window of 30 frames with a sampling rate of 8 would only capture around 3 s of the recording, making it impossible to capture one full breathing cycle. Taking this into account, both the width of the fusion kernel and the sampling rates of both branches were increased to enable RR estimation using a single convolutional model. A detailed analysis of the different configurations is presented in the Experimental Analysis section (Section 4).

### 3.2. Increased Receptive Field

In the study, the adjustment of various parameters was examined, including the receptive field of the kernels in the CNN backbone network. As mentioned previously, thermal images have less defined features than those captured in visible light due to heat flow in objects. To address this issue, this work evaluates different sizes of the receptive field. A larger receptive field allows the network to capture more global information about the input image, which can improve the network’s ability to recognize complex patterns and structures. For example, in image classification tasks, a larger receptive field can help the network recognize objects in the context of their surroundings and reduce the impact of noise in the input image. By considering a larger region of the input image, the network can average out small variations and focus on more significant features.

Since the backbone used in the SlowFast model is based on residuals, the estimation of the final receptive field is not straightforward because for *D* residual blocks there are $2^{D}$ possible paths from the input to the output. Following [39], if *d* skip connections are selected, the number of layers (*L*) in the CNN can be approximated as:

(1) L=(D−d)q

where *q* indicates the number of pathways in each block (usually 2). As described in [40], the effective receptive field (ERF) of the fully connected model can be approximated using the recurrence equation:

(2) $$ERF=\sum_{l=1}^{L}\left(\left(k_{l}-1\right)\prod_{i=1}^{l-1}s_{1}\right)+1$$

where *l* is the layer number, $k_{l}$ is the kernel size for this layer, and *s* is the stride size. Given (1), for simplicity of analysis, the ERF for different configurations of the proposed model can be compared by computing ERF (2) as a sum of (D−d)q layers, which in the biggest possible variant of the model (d=0) translates to Dq layers.

### 3.3. RR Estimation with Action Recognition Model

To make the base model suitable for the regression task, some necessary modifications were made to allow the model to predict continuous output values.

The first and most significant change involved replacing the final layer of the network, which in the original model used a softmax activation function to produce a probability distribution over classes. Working on a regression task, the final layer was replaced with a linear activation function. This allowed the model to predict continuous values instead of discrete classes.

In addition to changing the activation function, an appropriate loss function to measure the difference between the predicted values and the actual values has to be selected. Various loss functions were evaluated, including Mean Absolute Error (MAE), Mean Squared Error (MSE), Weighted MSE (WMSE) Mean, and Root Mean Squared Error (RMSE). The WMSE function was defined as:

(3) $$WMSE=\frac{1}{n}\sum_{i=1}^{n}\frac{w{\left(Y_{i}-{\hat{Y}}_{i}\right)}^{2}}{i}$$

where *w* is an adjustable parameter, $Y_{i}$ is prediction output, ${\hat{Y}}_{i}$ is the ground-truth label, and *n* is the number of samples. The intuition behind using the weighted MSE was to give more importance to prediction errors obtained for samples with the higher standard deviation, and thus to obtain the model that would be able to cover a wider range of RR values. Finally, once the architecture modifications and loss function selection were complete, the adapted model was trained on the RR regression task.

### 3.4. Dataset

Thermal imaging technology captures electromagnetic radiation intensities as precise digital values, usually with a 14-bit resolution. These digital values are then translated into temperature data by assigning color values to each one, which creates the final thermal image. To gather additional data for this research, a FLIR ONE^®^ thermal camera (Teledyne FLIR, Wilsonville, OR, USA) was utilized. It is a portable sensor that can be connected to a smartphone or tablet, making it suitable for use in various scenarios, such as remote medical diagnosis. The camera can capture images at an 8.7 Hz frame rate with a resolution of 80 × 60 pixels.

To validate the reliability of the proposed RESCUED model, a collection of 77 thermal video sequences was used. Of these sequences, 40 were taken from the SC3000 dataset [41], and the remaining 37 were gathered by us specifically for this research. Each sequence was 15 s in duration. The thermal video sequences were recorded with the participation of 46 volunteers. The average age of the volunteers was 36.8 ± 9.04, and the group consisted of 24 females and 22 males. The data collection was performed in a controlled environment with an average ambient temperature of 20 °C ± 1, with subjects facing the camera at a distance of 1.2 m in a seated position. To minimize any potential sources of noise that could affect the quality of the data, the volunteers were instructed to remain as still as possible during the data capture procedure. This was particularly important given the preliminary nature of this study and the need to obtain reliable baseline data for subsequent analysis. Volunteers also contributed by self-evaluating the number of breaths taken during each of the recordings. The resulting dataset consists of approximately 10,400 frames of thermal data, providing a valuable resource for developing and evaluating RR measurement algorithms based on thermal imaging. All volunteers provided their consent for using recorded data in the study.

## 4. Experimental Analysis

During the experimental analysis, various parameters were analyzed in a set of experiments to find the best-performing configuration. Initially, an extensive grid search was performed to select the model and training hyperparameters. The default configuration is presented in Table 1 along with the search space. In total, more than 600 runs were executed during the grid search process. After that, a detailed analysis of different parameters was done to confirm the claims of the proposed RESCUED solution, i.e., the need for the increased receptive field, the wider fusion kernel and adaptation of the sampling rates, and the influence of the loss function utilizing the modified network head. Therefore, only parameters corresponding to the network architecture were evaluated (network depth, receptive field size, fusion depth, and the sampling rate). In addition, the loss function, and the aggregation methods were also examined to ensure the correct behavior of the model when dealing with potential data outliers. The rest of the parameters corresponding only to the training procedure itself were set to the determined best values. In each experiment, one of the parameters was analyzed while keeping the others unchanged to allow for a fair evaluation of the influence of this parameter on the final model accuracy. Each configuration was evaluated on the set of 240 sequences extracted from eight recordings collected from eight different volunteers using the sliding window with 1 s overlap. There was no overlap among subjects between the train and the test set.

In the performed experimental analysis, the RMSE error was used for comparing the estimated RR with the ground-truth value, as shown in Equation (4).

(4) $$RMSE=\sqrt{\sum_{i=1}^{N}\frac{{\left(RR_{est}-RR_{gt}\right)}^{2}}{N}}$$

where *N* is the number of data recording, $RR_{est}$ is the estimated RR, and $RR_{gt}$ is the ground-truth RR. The reason for selecting this metric is that RMSE has the capability of amplifying large errors. This means it is useful when large errors are particularly undesirable, which is important in the performed study due to capturing marginal RR values that can indicate specifically important health conditions.

### 4.1. Evaluation of the Network Depth

In this step, the evaluation of different depths of the model backbone was performed. Given the sufficient training data, using deeper architectures should theoretically lead to better accuracy. The results obtained for ResNet50 and ResNet101 backbones are presented in Table 2.

As can be seen, the smaller model performed better, which may be the result of a small dataset and/or model overfitting (increasing variance).

### 4.2. Evaluation of the Receptive Field Size

Following [11], the increased receptive field might be beneficial when dealing with thermal data to make use of a more distant relationship between object features than in the visible light spectrum. Thus, the receptive fields of the feature extraction portion of the introduced model were increased. Please note that depth-wise convolutions remained unchanged. Table 3 presents a detailed summary of updated kernels. RMSE results obtained for each configuration are shown in Table 4.

When evaluated on the test set, the network trained with a larger receptive field (configuration 2) outperformed the one with a smaller receptive field (configuration 1) by 0.09, demonstrating the importance of receptive field size in improving the network’s performance in recognizing features in thermal images. On the other hand, creating a too large receptive field may not help either, which may be caused by insufficient training examples.

### 4.3. Evaluation of Fusion Depth and Sampling Rates

**Figure 4:**
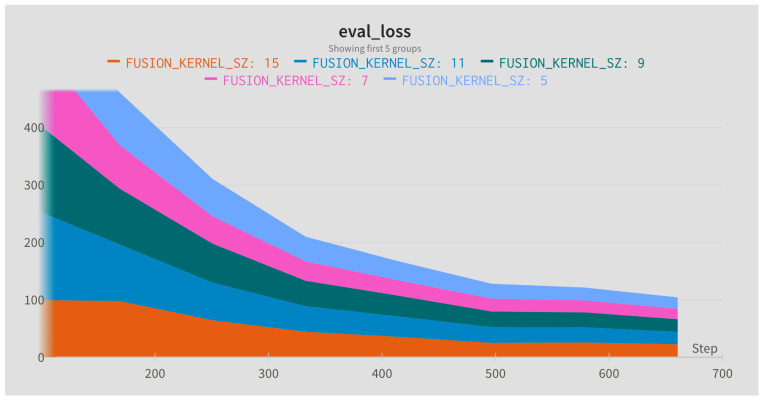
Evaluation loss for different sizes of the fusion kernel used for merging Slow and Fast paths.

**Figure 5:**
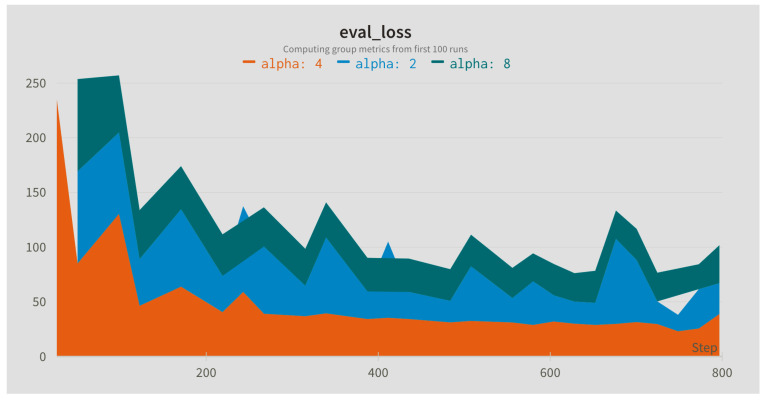
Evaluation loss for different values of the sampling frequency in the Slow path.

**Figure 6:**
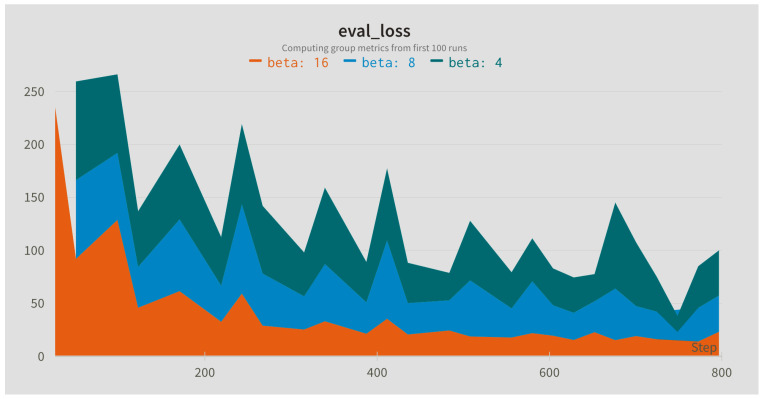
Evaluation loss for different values of the sampling frequency in the Slow path.

In this step, the influence of different sampling rates for the Slow (alpha) and Fast (beta) branches was compared. The sampling rates were applied to the input sequence with the default window length of 128 frames. In addition, different widths of the fusion kernel combining both paths were compared. Figure 4, Figure 5 and Figure 6 present evaluation loss achieved for different values of the fusion kernel used for merging both paths of the RESCUED model and different values of the sampling frequency used for Slow and Fast branches, respectively. Corresponding values of the error achieved on the test set are collected in Table 5, Table 6 and Table 7.

### 4.4. Evaluation of the Loss Function

In experiments, the impact of the choice of the loss function and reduction method (aggregating loss across samples in a batch) used during training on the final results was investigated. The results shown in Table 8 indicate that there was no significant impact on the test set performance for different aggregation techniques. On the other hand, the use of the MSE loss led to the lowest RR estimation error, which indicates that reducing outliers is crucial in the analyzed task and the use of absolute error (MAE) or smoothing introduced with RMSE may result in a worse performance. Nevertheless, it is worth noting that the impact of the loss function might differ for other hyperparameter settings. Thus, it is crucial to be mindful of the choice of the loss function and its potential influence on the performance of the model.

### 4.5. Comparison with Existing Methods

Finally, to ensure the reliability of the study, a five-fold cross-validation technique was employed to train and validate the model. The dataset is first divided into five parts of equal size. Subsequently, the model is trained on four of these parts, and the fifth part is kept aside for validation. The process of splitting data has a constraint of sorting recordings of the same volunteer in a single fold. This process is repeated five times to ensure that each part of the dataset is used as a validation set once. This technique helps us to assess the model’s performance more accurately and minimize the possibility of overfitting the training data. The achieved RMSE was averaged across all runs and compared with previous state-of-the-art methods (Table 9).

RRJR is a method proposed in [15] based on image and signal processing techniques. In the proposed method, the nostril area is manually marked and applied to all frames in the sequence. After that, the value per each frame is calculated as a mean value (or skewness) of pixel values within the marked nostril areas. The sequence of such values over time forms a signal which is then filtered and converted to the frequency domain. The dominant peak corresponds to the estimated RR value. HR means that the RRJR method was applied to the original high-resolution data. The methods Bicubic, DRESNet, TTSR, and EVM use bicubic interpolation, convolutional model DRESNet [11], Transformer model TTSR [42], and Eulerian Video Magnification [43] for improving the quality of sequences before applying the RRJR method, respectively. Our method (RESCUED) applied to original sequences outperformed all of the previous results, despite not using any image enhancement techniques to artificially boost the quality of the low-resolution thermal sequences.

## 5. Discussion

The presented work focused on evaluating the possibility of RR estimation from low-resolution thermal image sequences using a one-shot convolutional-based method. Such a solution possesses some important advantages over the previous methods that were based on a set of various image and signal processing algorithms offering only a semi-automated solution. First of all, according to the achieved results, the error of RR estimation was reduced. Secondly, the use of a fully convolutional-based method which does not require higher-precision signal analysis algorithms allows for its deployment on machine learning accelerators [44] supporting such topologies, thus making the model suitable for smart home solutions [45], driver monitoring systems in L2+ Advanced Driver Assistance Systems (ADAS) [46], surveillance or security solutions [47], other embedded edge use cases, and potentially for other vital signs as well [48]. Lastly, previous methods were able to achieve satisfactory performance when additional pre-processing algorithms were applied, i.e., super-resolution [11,41], motion magnification [49], denoising [24], and others. This is caused by the fact that thermal imagery usually has poor resolution compared to visible light data, and thus images have to be enhanced before estimating vital signs. However, the method proposed in this study achieves superior RR estimation accuracy without the need for the enhancement step, simplifying the overall process.

The performed in-depth analysis of various parameters of the model showed the importance of increasing the receptive field and fusion kernels when dealing with the thermal data which have different characteristics than visual light samples. This study aligns with similar research in the field [35]. It highlights the importance of carefully designing and selecting model topology based on the image spectrum for accurate predictions in neural networks targeting various image domains.

However, although the presented results are promising, they are only preliminary, and further work is required for supporting the presented findings. First of all, the respiration sequences used in the study were recorded only for normal breathing rates. It would be interesting to evaluate whether the model can be applied to abnormally slow or fast RR. Such a solution would potentially allow for detecting emergency situations, such as asthma, anxiety, pneumonia, congestive heart failure, or drug overdose, which would have a real practical value [50]. Such analysis will be performed in future work.

Another limitation of the presented RESCUED model is the large input size (two branches of a size 224 × 224 × 3 × 32 and 224 × 224 × 3 × 8) and the increased receptive fields, leading to more parameters and, as a result, a bigger model. Such a network may be difficult to deploy on resource-constrained devices with limited memory. Additionally, evaluated input window lengths of 64 and 128 combined with a sampling rate of 3 may introduce additional latency to fill up the input buffer. While all methods used to evaluate RR need to accumulate sequence data in a buffer before extracting a signal, these parameters should be taken under consideration, especially when using the model to estimate RR from a live stream. Taking it into account, the future work will focus on reducing the network size by using smaller inputs and deployment of better techniques for increasing the receptive field, i.e., atrous convolutions [51], or depth-separable convolutions [52]. It would be also important to analyze the influence of motion on the quality of the extracted signal [53,54].

## 6. Conclusions

The presented work introduces a novel approach to respiratory rate estimation, one of the key parameters in patient monitoring. The RESCUED method uses a robust machine learning algorithm with two concurrent pathways (Slow and Fast branches) to accurately estimate the respiratory rate from thermal sequences captured by a small form factor thermal camera. Combination of two paths covering expandable depths of thermal data for vital sign estimation has not been done in the literature before. The experimental results demonstrate that the proposed innovative approach outperforms the state-of-the-art methods in terms of accuracy and robustness, eliminating the need for converting data to the frequency domain, which might not be supported on ML accelerators. Thus, the proposed method has great potential for deployment in real-world scenarios, such as hospital and home-based patient monitoring. Further research could investigate the feasibility of integrating our approach with wearable devices and developing real-time monitoring systems to aid in the early detection and treatment of respiratory disorders. Overall, this work provides a promising direction for improving the accuracy and reliability of respiratory rate monitoring.

## Figures and Tables

**Table 1:** Hyperparameter search space used to identify the best-performing configuration of the RESCUED model.

Parameter	Best Value	Search Space
lossfunc	MSE	MSE, RMSE, WMSE
reduction	mean	mean, max
samplingrate	3	2, 3, 4, 5
input window length	128	64, 128
alpha	4	2, 4, 8
beta	16	4, 8, 16
fusionkernels	15	5, 7, 9, 11, 15
warmupepoch	64	32, 64, 96
warmuplr	$5\times 10^{-5}$	$1\times 10^{-4}$, $3\times 10^{-4}$, $5\times 10^{-4}$
		$7\times 10^{-4}$, $9\times 10^{-4}$, $5\times 10^{-5}$
dropout	0.2	0.2, 0.4, 0.6
target_fps	8	8, 30
backbone	ResNet50	ResNet50, ResNet101
receptive field	5 × 5	3 × 3, 5 × 5, 7 × 7, 9 × 9

**Table 2:** Root Mean Square Error (RMSE) comparison between different depths of the feature extraction model used in the backbone of the proposed model. The best result is marked in blue.

Backbone Depth	RMSE (Test Set)
ResNet50	1.51 ± 0.98
ResNet101	1.64 ± 1.09

**Table 3:** SlowFast kernels updated in the proposed RESCUED model to increase the receptive field and better match the characteristics of thermal data. Used notation: temporal kernel size × spatial kernel size, kernel filters, e.g., 1 × $5^{2},\;64$ means 64 kernels of a size 1 × 5 × 5. Configuration 1 marked in red, configuration 2 marked in cyan, configuration 3 marked in olive.

Stage	Slow Path	Fast Path
Conv 1 Temp	1 × $5^{2}/7^{2}/9^{2},64$	5 × $5^{2}/7^{2}/9^{2},8$
Residual 1	1 × $1^{2},64$	1 × $3^{2}/5^{2}/7^{2},8$
	1 × $3^{2}/5^{2}/7^{2},64$	1 × $1^{2},256$
	3 × $1^{2},8$	1 × $1^{2},32$
Residual 2	1 × $1^{2},64$	1 × $3^{2}/5^{2}/7^{2},8$
	1 × $3^{2}/5^{2}/7^{2},64$	1 × $1^{2},256$
	3 × $1^{2},8$	1 × $1^{2},32$
Residual 3	1 × $1^{2},64$	1 × $3^{2}/5^{2}/7^{2},8$
	1 × $3^{2}/5^{2}/7^{2},64$	1 × $1^{2},256$
	3 × $1^{2},8$	1 × $1^{2},32$
Residual 4	1 × $1^{2},64$	1 × $3^{2}/5^{2}/7^{2},8$
	1 × $3^{2}/5^{2}/7^{2},64$	1 × $1^{2},256$
	3 × $1^{2},8$	1 × $1^{2},32$

**Table 4:** RMSE comparison between different sizes of the receptive field of the backbone of the proposed model. Blue—first best, orange—second best. ERF (2) has been calculated for modified Slow and Fast branches and takes only spatial resolution.

Backbone Receptive Field	ERF	RMSE (Test Set)
Configuration 1	37	1.51±0.98
Configuration 2 (original)	71	1.42±0.92
Configuration 3	105	1.49±0.94

**Table 5:** Root Mean Square Error (RMSE) comparison between different depths of the fusion kernel. Blue—first best, orange—second best.

Fusion Kernel Depth	RMSE (Test Set)
5 (original)	1.61±1.06
7	1.64±1.09
9	1.55±0.92
15	1.46±0.92

**Table 6:** RMSE comparison between different values of the sampling rate of the Slow path. Blue—first best, orange—second best.

Slow (Alpha) Sampling Rate	RMSE (Test Set)
2 (original)	1.68±0.89
4	1.55±1.02
8	1.69±0.90

**Table 7:** RMSE comparison between different values of the sampling rate of the Fast path. Blue—first best, orange—second best.

Fast (Beta) Sampling Rate	RMSE (Test Set)
4 (original)	1.68±0.89
8	1.65±0.89
16	1.55±0.95

**Table 8:** RMSE comparison between different loss functions used during training. MEAN and MAX represent the aggregation methods for each sample in the batch. Blue—first best, orange—second best.

Loss Function	RMSE (Test Set)
MSE Max	1.44±0.98
MSE Mean	1.44±0.99
MAE Max	1.64±0.97
MAE Mean	1.64±1.01
RMSE Max	1.52±0.98
RMSE Mean	1.52±0.98
WMSE Max	1.61±0.96
WMSE Mean	1.62±0.97

**Table 9:** Root Mean Square Error (RMSE) comparison between various methods for RR estimation. Our proposed method outperforms all presented methods and is also the most consistent. Blue—first best, orange—second best.

Method	RMSE (Test Set)
RRJR + HR	1.68±1.71
RRJR + Bicubic	3.60±4.83
RRJR + DRESNet	1.76±1.90
RRJR + EVM	3.42±4.47
RRJR + TTSR SingleT	2.54±3.69
RRJR + TTSR MultiT	2.40±3.56
RRJR + TTSR SingleVL	1.91±2.16
Ours (RESCUED) + HR	1.46±0.92

## Data Availability

The data presented in this study are available on request from the corresponding author. The data are not publicly available due to privacy.
